# Income disparity in school readiness and the mediating role of perinatal maternal mental health: a longitudinal birth cohort study

**DOI:** 10.1017/S204579602000102X

**Published:** 2021-01-08

**Authors:** E. C. Law, R. Aishworiya, S. Cai, A.-A. Bouvette-Turcot, B. F. P. Broekman, H. Chen, L. M. Daniel, P. D. Gluckman, L. P. C. Shek, S. K. H. Tay, Y. S. Chong, G. C.-H. Koh, M. J. Meaney

**Affiliations:** 1 Department of Paediatrics, Yong Loo Lin School of Medicine, National University of Singapore, Singapore, Singapore; 2 Khoo Teck Puat-National University Children's Medical Institute, National University Health System, Singapore, Singapore; 3 Singapore Institute for Clinical Sciences (SICS), Agency for Science, Technology and Research (A*STAR), Medical Drive, Singapore, Singapore; 4 Douglas Mental Health University Research Centre, McGill University, Montreal Quebec, Canada; 5 Department of Psychiatry, VU University Medical Centre, Amsterdam, The Netherlands; 6 Department of Psychological Medicine, KK Women's and Children's Hospital, Singapore, Singapore; 7 Duke-NUS Medical School, Singapore, Singapore; 8 Department of Child Development, KK Women's and Children's Hospital, Singapore, Singapore; 9 Liggins Institute, University of Auckland, Grafton, Auckland, New Zealand; 10 Department of Obstetrics and Gynaecology, National University Health System and National University of Singapore Yong Loo Lin School of Medicine, Singapore, Singapore; 11 Saw Swee Hock School of Public Health, Tahir Foundation Building, National University of Singapore, Singapore, Singapore; 12 Department of Psychiatry, Ludmer Centre for Neuroinformatics and Mental Health, McGill University, Montreal Quebec, Canada

**Keywords:** Household income, maternal mental health, perinatal mood, school readiness

## Abstract

**Aims:**

There is compelling evidence for gradient effects of household income on school readiness. Potential mechanisms are described, yet the growth curve trajectory of maternal mental health in a child's early life has not been thoroughly investigated. We aimed to examine the relationships between household incomes, maternal mental health trajectories from antenatal to the postnatal period, and school readiness.

**Methods:**

Prospective data from 505 mother–child dyads in a birth cohort in Singapore were used, including household income, repeated measures of maternal mental health from pregnancy to 2-years postpartum, and a range of child behavioural, socio-emotional and cognitive outcomes from 2 to 6 years of age. Antenatal mental health and its trajectory were tested as mediators in the latent growth curve models.

**Results:**

Household income was a robust predictor of antenatal maternal mental health and all child outcomes. Between children from the bottom and top household income quartiles, four dimensions of school readiness skills differed by a range of 0.52 (95% Cl: 0.23, 0.67) to 1.21 s.d. (95% CI: 1.02, 1.40). Thirty-eight percent of pregnant mothers in this cohort were found to have perinatal depressive and anxiety symptoms in the subclinical and clinical ranges. Poorer school readiness skills were found in children of these mothers when compared to those of mothers with little or no symptoms. After adjustment of unmeasured confounding on the indirect effect, antenatal maternal mental health provided a robust mediating path between household income and multiple school readiness outcomes (*χ*^2^ 126.05, df 63, *p* < 0.001; RMSEA = 0.031, CFI = 0.980, SRMR = 0.034).

**Conclusions:**

Pregnant mothers with mental health symptoms, particularly those from economically-challenged households, are potential targets for intervention to level the playing field of their children.

## Background

Socioeconomic status (SES) has profound effects on the capacity and achievement of children, including cognitive, social–emotional and brain development (Hackman *et al*., 2010; Shonkoff *et al*., 2012; Piccolo *et al*., 2016; Sheridan and McLaughlin, 2016). SES-related contextual factors are also salient risks for suboptimal maternal mood (Petterson and Albers, 2001; Kiernan and Huerta, 2008; Allen *et al*., 2014). While prior studies have largely focused on demonstrating the negative sequelae of low SES, recent studies use a life course approach to study the familial transmission of disadvantage and to describe potential explanatory pathways (Cents *et al*., 2013; Pearson *et al*., 2013).

Maternal mental health has been proposed as a candidate pathway contributing to SES disparity in multiple child developmental outcomes, yet studies confirming this hypothesis are lacking. There is, however, plentiful and compelling evidence for the association between maternal mental health and developmental competencies of the offspring (Cents *et al*., 2013; Pearson *et al*., 2013; O'Donnell and Meaney, 2017; Meaney, 2018). Recently, our own group and others have shown that the experience of maternal depression and anxiety not only ‘gets under the skin’ of children, but that discernible differences are already evident in the brains of newborn infants after antenatal exposure as a foetus (Qiu *et al*., 2013; Noble *et al*., 2015; Rifkin-Graboi *et al*., 2015; Lebel *et al*., 2016; Shen *et al*., 2016; Wen *et al*., 2017).

During pregnancy, 10–15% of women report depression and anxiety symptoms above clinical cut-offs, while up to 30% have high, but often subclinical, levels of symptoms (Meaney, 2018). Perinatal mental health studies often focus on women meeting criteria for a clinical disorder, which may miss the latter group of women. Mothers with high symptom counts, although not at clinical levels, transmit increased risks related to psychological outcomes to the next generation when compared to mothers without symptoms (Weinberg *et al*., 2001; Tietz *et al*., 2014; Meaney, 2018). Consequently, studying depressive and anxiety symptoms instead of clinical diagnoses enables the understanding of whether child outcomes vary based on different degrees of maternal symptoms.

During the perinatal period, depressive and anxiety symptoms covary and are hard to isolate independently (Lancaster *et al*., 2010; Verreault *et al*., 2014). For example, depressive symptoms at the second trimester are correlates of high anxiety levels at the third trimester, which in turn predict depressive symptoms in the postnatal period (Skouteris *et al*., 2009). As perinatal maternal symptoms vary in timing, severity and duration, it is best studied using trajectories across time (Dennis *et al*., 2017). Prior studies generally examine either antenatal or postnatal period while controlling for the other, which do not fully capture the dynamic processes across time points (Goodman and Tully, 2009; Pearson *et al*., 2013). Hence, this present study incorporates maternal depressive and anxiety symptom trajectories from the antenatal to postnatal period to represent a whole experience for the child using latent growth curve models (LGCMs).

As school readiness constitutes a critical skill set for shaping children's long-term life successes and health, our group seeks to understand whether maternal mental health trajectory forms an underlying pathway explaining why children are differentially affected by poverty. School readiness is a multifaceted concept that encompasses skill sets needed for preparation into school and wider ecological systems (High, 2008; Moreno, 2013; Scharf, 2016). By this definition, school readiness skills stretch beyond general knowledge and pre-academic skills, such as literacy and early mathematics, and include additional dimensions (e.g. behavioural and socio-emotional, cognitive and executive functions) (Welsh *et al*., 2010; Blair and Raver, 2015; Perry *et al*., 2018). Four dimensions of school readiness skills are the outcomes in this study and are captured from 2 to 6 years of age.

Mediators of the income-school readiness gap, especially measurable and modifiable ones, are potential leverage points that may inform interventions. In this study, we targeted perinatal maternal mental health as a promising mediator based on our group's neuroimaging and neurophysiological findings (Qiu *et al*., 2013; Rifkin-Graboi *et al*., 2015; Wen *et al*., 2017). We hypothesised that antenatal maternal mental health and its trajectory from pregnancy to 2-years postpartum, both attenuated the effects of household income on child school readiness.

## Methods

### Participants

**Fig. 1.**
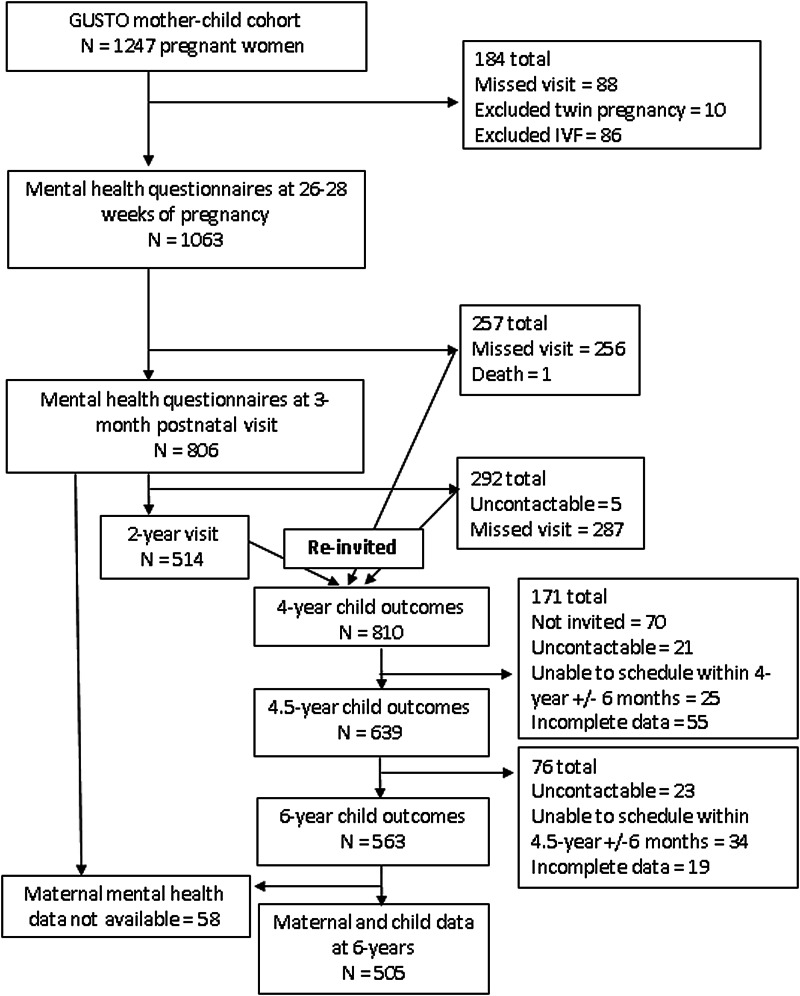
Study flow diagram.

Data were obtained from the Growing Up in Singapore towards Healthy Outcomes (GUSTO) longitudinal birth cohort study (Soh *et al*., 2014). The cohort consisted of 1247 mother–child dyads who were representative of the multi-ethnic population in Singapore. Pregnant women were recruited in their first trimester in 2009 to 2010 from two public hospitals in Singapore. Women with preterm infants (<37 weeks' gestation), twin pregnancy or conception via *in vitro* fertilisation techniques were excluded from this study. Participants were followed through and beyond delivery and their children were seen for neurocognitive testing regularly either in participants' home or at our neurodevelopmental research centre (Fig. 1). At 4 years of age, we re-invited families who missed a prior postnatal visit to keep their enrolment active. As our cohort consisted of many study domains, at 4.5 years, we prioritised our invitation to families who had attended the most postnatal visits between 6-months and 4-years to match our laboratory capacity, reduce the time burden, and allow for the most complete set of data for analysis. Not all our instruments produced age-equivalent scores; therefore, participants who could not be scheduled within 6 months of the designated visit time were not included in the analyses.

### Exposures

#### Household income

Household income obtained during pregnancy was in four groups (<S$2000, $2000–4000, $4000–6000 and >$6000/month; S$1 = US$0.73 = £0.57). There is currently no official poverty line or income-to-needs index in Singapore. However, an absolute household monthly income of ~S$2000 is often used to represent those who satisfy the household income criterion for a governmental financial subsidy (Donaldson *et al*., 2013). Hence, based on the context of this birth cohort, the decision was made to use an income cut-off <S$2000 *v*. ⩾$2000 per month in inferential statistics to allow for real-world relevance.

#### Maternal mood

The following two measures were administered between 26 and 28 weeks of pregnancy and also at 3- and 24-months postpartum. *Edinburgh Postnatal Depression Scale (EPDS)*. The EPDS is a widely-used questionnaire that assesses the presence and severity of depressive symptoms (Cox *et al*., 1987). Initially, it was developed for postnatal depression and was subsequently validated for depression during pregnancy (Murray and Cox, 1990). Although the cut-off of ‘above 12’ (i.e. 13 or more) is used to screen for clinical depression in a general population, the cut-off during pregnancy is ‘above 14’ (i.e. 15 or more) (Matthey *et al*., 2006). It is important to note that this tool also elicits anxiety symptoms (e.g. I have been anxious or worried for no good reason, I have felt scared or panicky). *State-Trait Anxiety Inventory (STAI)*. The STAI is a well-validated measure of state and trait anxiety used in perinatal studies to assess maternal mood (Spielberger, 2010). We used state anxiety to represent temporal mood fluctuations during pregnancy. As STAI does not have an established cut-off for subclinical symptoms, we first determined the extent, in standard deviations, to which the EPDS subclinical cut-off range (EPDS 9–14) was above the cohort mean for depressive symptoms, then we applied the same calculations to determine a cut-off range for anxiety symptoms.

### Four dimensions of school readiness

The following seven assessments measured different dimensions of school readiness, namely behavioural and socio-emotional, pre-academic, fluid reasoning and working memory, a component of executive functions. These were the same assessments that were validated against the cohort's school-age data (unpublished) and were previously used by other longitudinal cohorts as a school readiness panel (O'Donnell *et al*., 2014). As English is the medium used in education in Singapore, tools administered to children were in English. *Child Behaviour Checklist, Preschool (CBCL) at age 2*. The CBCL is a validated questionnaire for behavioural and socio-emotional problems in children ages 1.5–5, is widely used in mental health services, and validated in Singapore (Achenbach and Rescorla, 2000; Woo *et al*., 2007). We used the internalising and externalising raw scores, as recommended by the Achenbach manual, to capture two spectrums of behavioural difficulties (Achenbach and Rescorla, 2000). *Lollipop Test.* The Lollipop is a measure of general knowledge and comprises four subtests: colours and shapes, numbers, letters and spatial recognition (Chew, 1981). The Lollipop has been demonstrated to have good reliability and predicts performance in fourth grade (Chew, 1981). Number *Knowledge Test (NKT)*. The NKT consists of questions testing numerical concepts and counting skills of the child. The NKT has good validity and is representative of numeracy in kindergarten (Griffin, 2002). *Peabody Picture Vocabulary Test, 4th Edition (PPVT-IV).* The PPVT-IV is a reliable instrument measuring receptive vocabulary in individuals from ages 2.5 to 90, with reliability and validity coefficients above 0.90 (Naglieri and Pfeiffer, 1983). *Comprehensive Test of Phonological Processing (CTOPP)*. The CTOPP is a measure of phonological processing abilities, which are crucial for reading and achievement, and has internal and test-retest reliability coefficients above 0.80 (Wagner *et al*., 2013). *Kaufman Brief Intelligence Test, Second Edition (KBIT-2) at age 4.5*. The non-verbal score of the KBIT-2 is a measure of fluid reasoning (i.e. problem-solving and identifying patterns) for individuals ages 4–90 and has a correlation coefficient of 0.88 (95% CI: 0.74–0.95) with the matrix reasoning score of the Wechsler Intelligence Scale for Children, Fourth Edition (Kaufman and Kaufman, 2004). *Cambridge Neuropsychological Test Automated Battery (CANTAB) Spatial Working Memory (SWM) at age 6*. The CANTAB is a well-validated, computerised battery that covers a range of executive function tasks (Luciana and Nelson, 2002). The SWM in the CANTAB tests children's ability to retain spatial information mentally. We used the number of total errors (i.e. the sum of between and within errors) from the 4, 6 and 8 box SWM tasks as the outcome (Fried *et al*., 2015).

### Statistical analysis

To compare characteristics of children from various household income groups, we conducted a one-way analysis of variance (ANOVA) with 3000-iteration bootstrapping and Chi-square for the trend test with an odds ratio on the interval and categorical variables, respectively. We then determined the school readiness gap between children from the bottom and the top household income groups using mean differences in standard deviations (*Z*-scores). We also utilised one-way ANOVA and *post-hoc* Tukey to compare the child outcomes between groups of mothers with (1) clinical levels, (2) high, but subclinical levels and (3) little or no antenatal depressive or anxiety disturbances. Descriptive analyses were completed using IBM SPSS version 22.0 (Armonk, NY: IBM Corp).

Since both questionnaires (i.e. STAI and EPDS) collectively measured different facets of mental health and at the same time contained overlapping items, we first tested whether depressive and anxiety symptoms, as manifest variables, were representative of one or two mental health constructs (see online Supplementary text). Similar to an exploratory bi-factor analysis completed by our group previously, depressive symptoms (loadings 0.84–0.93) and anxiety symptoms (0.80–0.87) both contributed comparably to one general mental health construct (see online Supplementary Fig. S1) (Phua *et al*., 2017). Hence, we created a single mental health trajectory using latent constructs at three time points: antenatal mental health (26–28 weeks' gestation), Time 1 (postnatal 3 months) and Time 2 (postnatal 24 months).

Next, we examined the linear trajectory of maternal mental health from these time points and its relation with child outcomes. LGCMs were most suited in this case to capture an individual's mood trajectory and inter-individual differences in these trajectories. Finally, we added household income as an exposure and school readiness skills as outcomes into the LGCMs to understand the contribution of antenatal maternal mental health (intercept) and its linear trajectory (slope) as potential mediators. As the indirect paths through two covarying latent mediators could be sensitive to omitted confounders, we conducted correlated augmented model sensitivity analysis (CAMSA), which used correlations between residuals, termed confounder correlations, to model the effects of the omitted confounders (Tofighi *et al*., 2019). These models were completed using Mplus 8 (Los Angeles, CA: Muthén & Muthén). *P*-values were provided in multiple degrees of freedom tests, including LGCMs. For all other analyses, bootstrap confidence intervals were presented.

### Missing data

Prior to analysis, we estimated that the minimum sample size needed for our final LGCM to detect an effect (Cohen's *d* of 0.25) was 306. A *post-hoc* Monte Carlo simulation was also conducted with our sample size to understand whether the observed and expected Chi-square tests of model fit were comparable at *α* = 0.05 and to ensure that *β* was >0.80. Our *post-hoc* Monte Carlo simulation with a sample size of 505 resulted in powers, as expressed in % significant coefficients, of 0.84–1.00. Given that the indicator was categorical, we utilised robust maximum likelihood (MLR) estimation to account for 49 children who attended the 6-year-old visit but missed the 2-year-old visit.

### Ethics approval

The study was approved by the Singapore National Healthcare Group and SingHealth Group Institutional Review Boards. All mothers gave informed consent at recruitment and after each amendment, and all children, who turned 7 years in 2017, assented to the study.

## Results

**Fig. 2.**
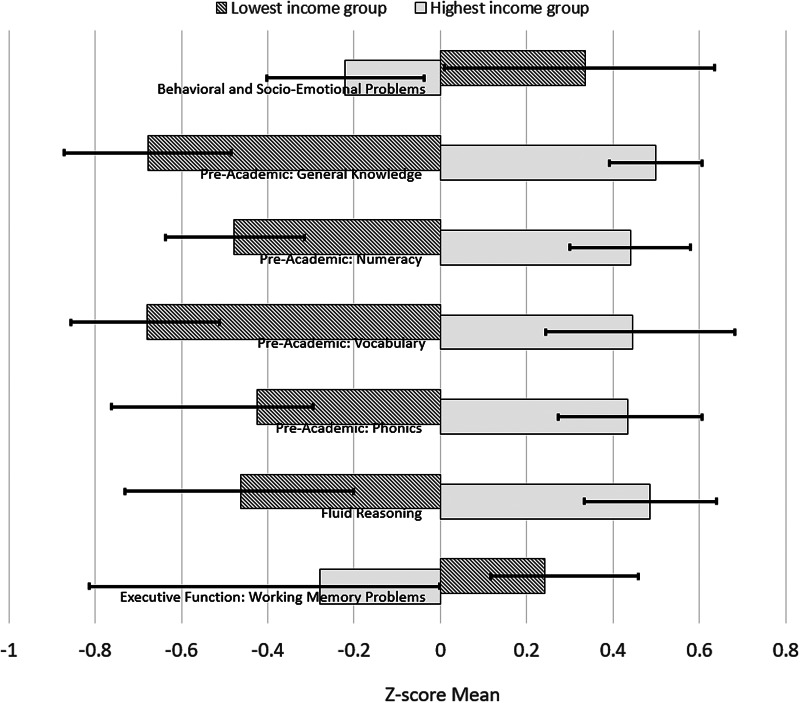
Differences in children's outcomes between the top and bottom household income groups. Whiskers represent 95% bias-corrected confidence interval of the *Z*-score mean.

Table 1 shows the demographic characteristics of the cohort. Sixteen percent of the mothers reported a household income <S$2000/month (US$1470/month or £1133/month). Between household income groups, notable gradients were found in maternal mental health and child outcomes. Following the format of the Programme for International Student Assessment (PISA), Fig. 2 shows the *Z*-score mean differences in child outcomes between the top and the bottom household income groups (Organisation for Economic Co-operation Development, 2012). We found that the poverty-related gaps were 0.60 s.d. (95% Cl: 0.28–0.92) in the behavioural and socio-emotional dimension, 0.86 (95% Cl: 0.64, 1.07) to 1.21 s.d. (95% CI: 1.02, 1.40) in major pre-academics (i.e. general knowledge, numeracy, vocabulary and phonics), 0.95 s.d. (95% CI: 0.66, 1.23) in fluid reasoning and 0.52 (95% Cl: 0.23, 0.67) in working memory.

**Table 1.** Demographics and characteristics of the cohort (by household income level)

	Cohort *N* = 1076		<S$2000 *n* = 177		S$2000–$3999 *n* = 338		S$4000–$5999 *n* = 271		>S$6000 *n* = 290		OR (95% CI) ⩾ S$2000 relative to < S$2000
	*n*	%	*n*	%	*n*	%	*n*	%	*n*	%	
*Maternal education*											
High school and below (Ref)	353	32.1	114	66.3	151	45.8	59	22.3	12	4.4	NA
Post high school	398	36.2	50	29.1	146	44.2	121	45.8	62	22.5	(2.65, 5.27) 3.74
University	348	31.7	8	4.7	33	10.0	84	31.8	201	73.1	(13.03, 55.83) 26.97
Male (Ref)	537	52.7	78	48.1	168	52.0	133	52.2	158	56.6	(0.59, 1.13) 0.81
Breastfeeding >3months	582	54.1	51	28.8	143	42.3	160	59.0	228	78.6	(2.54, 5.56) 3.76
Smoke exposure (cotinine level and/or reported)	160	14.9	51	28.8	68	20.1	37	13.7	9	3.1	(0.24, 0.51) 0.35
	Mean	s.d.	Mean	s.d.	Mean	s.d.	Mean	s.d.	Mean	s.d.	*p*
*Maternal*											
Age at delivery	31.41	5.10	29.67	6.14	30.72	5.35	31.32	4.69	31.41	5.10	<0.001
Parity (antenatal)	0.84	0.95	1.14	1.07	0.96	1.05	0.74	0.88	0.65	0.79	<0.001
Gestational age	38.78	1.44	38.62	1.44	38.75	1.43	38.61	1.28	39.04	1.56	0.002
*Antenatal*											
Depression score (EPDS)	7.45	4.46	8.81	5.03	8.06	4.27	7.21	4.45	6.01	3.89	<0.001
State-anxiety score (STAI-S)	34.72	9.79	38.83	10.01	36.71	9.50	33.34	9.17	31.27	9.15	<0.001
Total anxiety score (Total STAI)	71.26	17.89	78.72	18.55	74.77	17.09	69.03	16.39	65.01	16.92	<0.001
*Postnatal 3-month*											
Depression score (EPDS)	6.48	4.76	7.74	4.90	7.24	5.14	6.17	4.47	5.26	4.29	<0.001
State-anxiety score (STAI-S)	34.17	10.28	37.51	10.05	35.45	10.55	33.52	10.07	31.37	9.58	<0.001
Total anxiety score (Total STAI)	70.54	19.34	76.69	18.65	72.87	20.10	69.33	18.66	65.23	18.39	<0.001
	Cohort		<S$2000 (Ref.)		S$2000–$3999		S$4000–$5999		>S$6000		
	Mean	s.d.	Mean	s.d.	Mean	s.d.	Mean	s.d.	Mean	s.d.	
*Postnatal 24-month*											
Depression score (EPDS)	6.60	4.97	8.15	4.65	7.42	5.18	6.10	5.19	5.71	4.75	0.004
State-anxiety score (STAI-S)	34.73	10.00	37.95	9.10	36.29	9.93	34.41	10.09	32.25	10.24	<0.001
Total anxiety score (Total STAI)	70.97	19.55	77.22	17.17	74.19	19.08	70.52	20.35	66.03	20.05	<0.001
*Child*											
Birthweight	3.12	0.45	3.06	0.44	3.08	0.44	3.09	0.46	3.17	0.45	0.047
*Four dimensions of school readiness*											
*(1) Behavioural and social–emotional at 2 years, Z-score*											
Internalising	0.00	1.00	0.559	1.22	0.11	1.09	−0.10	0.83	−0.32	0.79	<0.001
Externalising	0.00	1.00	0.350	1.11	0.11	1.01	−0.06	0.92	−0.20	0.96	0.004
*2) Pre-academic skills at 4 years, Z-score*											
Lollipop	0.00	1.00	−0.61	0.99	−0.28	0.99	0.10	0.90	0.46	0.81	<0.001
NKT	0.00	1.00	−0.53	0.88	−0.28	0.87	0.04	0.84	0.44	1.08	<0.001
PPVT	0.00	1.00	−0.66	0.81	−0.34	0.82	0.20	0.97	0.43	1.02	<0.001
CTOPP	0.00	1.00	−0.42	0.69	−0.27	0.67	0.05	0.93	0.43	1.25	<0.001
*3) Fluid reasoning at 4.5 years, Z-score*	0.00	1.00	−0.46	1.07	−0.25	0.92	0.06	0.94	0.46	0.93	<0.001
*4) Executive function (working memory) at 6 years*											
SWM total errors	75.16	14.46	76.52	12.79	77.72	15.37	74.60	14.62	71.93	13.56	0.010

**Fig. 3.**
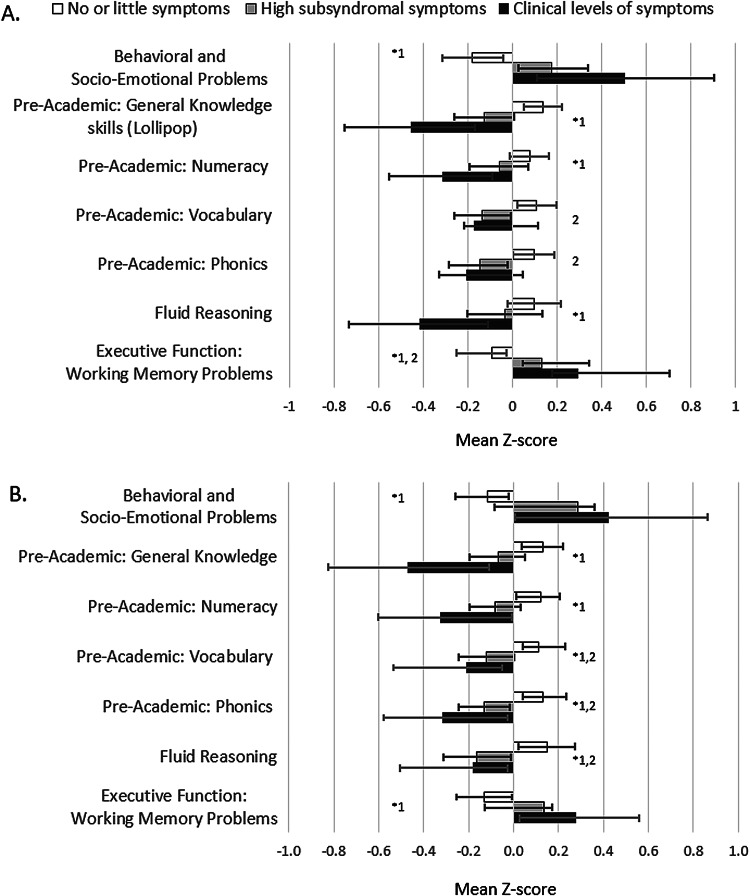
Child outcomes by gradients of (A) maternal depressive and (B) anxiety symptoms. ANOVA *p < 0.05 and *Post-hoc* Tukey *p* < 0.05 ^1^no vs. clinical, ^2^no vs. high.

Of the 7.3 and 6.5% of mothers reporting clinical levels of depressive and anxiety symptoms, respectively, at least 78.6% reported both types of symptoms. Another 30.9 and 31.4% were in the highest, subclinical depressive and anxiety (>0.35 and >1.50 s.d. above the mean of EPDS and STAI) levels of symptoms, respectively. There was a depressive symptom gradient seen in most of the child outcomes, except for vocabulary and phonics; however, the state anxiety gradient was present for all outcomes (Fig. 3).

Our initial step in building an LGCM was to utilise different Latent State models to determine whether the latent maternal mental health constructs from the two depression and anxiety scales at three time points were valid and to understand the residual structure of the models (see online Supplementary text, online Supplementary Figs S1, S2 and S3). Online Supplementary Table S1 (see online Supplementary material) shows the improvements in the fit indices with each new model. The evaluation of model fit was based on the Chi-square statistic, comparative fit index (CFI), root mean square error of approximation (RMSEA), standardised root mean square residual (SRMR) and Akaike information criteria (AIC). It is important to note that a non-significant result in the Chi-square statistic of latent models is known to be difficult to achieve in large samples. The most parsimonious model with CFI >0.95, RMSEA <0.05 and SRMR 0.05 was selected.

From our initial LGCM (see online Supplementary Fig. S4), we found that pregnant women in this cohort varied substantially in their antenatal mental health (*Var*_INTERCEPT_ = 12.13, *Z* = 11.54, *p* < 0.001) and in their intra-individual change of mental health over time (*Var*_SLOPE_ = 1.13, *Z* = 3.21, *p* = 0.001). However, as a group, a general pattern emerged in that the mean change in mental health over time was small and negative (*M*_SLOPE_ = −0.21, *Z* = −3.60, *p* < 0.001), indicating that mothers reported an overall improved mental health from antenatal to the postnatal period. The negative correlation of −0.167 in online Supplementary Fig. S4 (see online Supplementary material) between the intercept and the slope indicated that mothers with poorer antenatal mental health symptoms tended to show fewer improvements over time when compared to mothers with more optimal antenatal mental health.

**Fig. 4.**
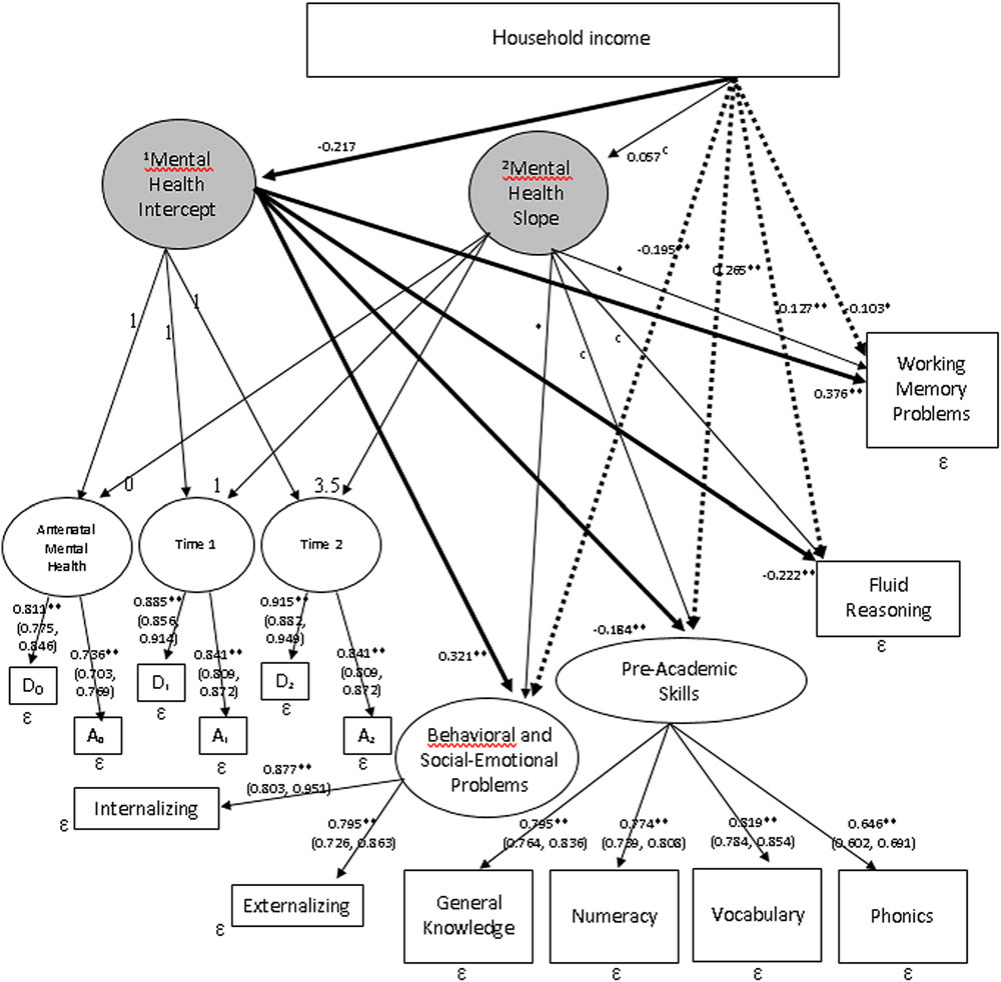
LGCMM with predictor and outcomes. Rectangular: observed variable; oval: latent variable; *p < 0.05; **p < 0.01; 95% CIs are shown in parentheses; ^1^Latent variable derived from the two depressive and anxiety symptom measures during pregnancy (time intercept = 0); ^2^Trajectory of the latent mental health variables from pregnancy, to postnatal 3-months and 24-months. Solid lines represent indirect paths and dashed lines represent direct paths to the outcomes.

We next sought to understand whether household income influenced mental health trajectories and child outcomes, and subsequently whether antenatal maternal mental health provided an indirect path to predict child outcomes. Figure 4 shows our final latent growth curve mediation model (LGCMM). The higher the household income, the better the antenatal maternal mental health (*β* = −0.217, *p* < 0.001) and all four dimensions of school readiness skills (Table 2 direct path coefficients). Furthermore, antenatal maternal mental health mediated at least a quarter of the total effects of household income on the offspring's behavioural and social–emotional skills at 2 years as well as fluid reasoning at 4.5 years, 13.2% of the total effects on pre-academic skills at 4 years and 44.2% of the total effects on working memory at 6 years (Table 2).

**Table 2.** Path estimates of the direct effects and indirect effects via the mental health intercept mediator (*i*)

Paths from household income to	Direct	Indirect to *i*	Indirect from *i*	*p* (*x* to *y* via *i*)	% of indirect/total
Behavioural and social–emotional problems	−0.195 (−0.289, −0.102)	−0.217 (−0.283, −0.150)	0.321 (0.221, 0.421)	<0.001	26.4
Pre-academic skills	0.265 (0.200, 0.327)		−0.184 (−0.269, −0.095)	0.007	13.2
Fluid reasoning	0.127 (0.048, 0.203)		−0.222 (−0.312, −0.135)	0.001	27.5
Working memory problems	−0.103 (−0.201, −0.013)		0.376 (0.309, 0.465)	<0.001	44.2

No indirect effects found via the mental health slope mediator; total effects = sum of direct effect and indirect effect (product of indirect to *i* and indirect from *i*).

Maternal symptom trajectory provided paths to behavioural and social–emotional as well as working memory problems in this study; however, these changes over time were not predicted by household income and could not provide a complete indirect path to these two outcomes. A correlated augmented model designed to quantify the unmeasured confounder bias in models with intercept and slope mediators was therefore not required. Instead, sensitivity plots with 95% Cl were produced to understand the extent to which the unmeasured confounders could reverse the mediation results of the LGCMM (Imai *et al*., 2010; VanderWeele, 2016; VanderWeele and Tchetgen Tchetgen, 2017). We plotted our indirect effects against a range of hypothetical sensitivity parameters, also called rho (see online Supplementary Fig. S5). The plots indicated that the indirect paths to the four school readiness dimensions would be trustworthy until the sensitivity parameters were 0.70, 0.40, 0.43 and 0.64, respectively, which were highly implausible given the actual coefficients listed in Fig. 4. Hence, the sensitivity analyses indicated that antenatal mental health was a robust mediator between household income and school readiness, even when unmeasured confounders were accounted for.

## Comment

### Principal findings

This longitudinal birth cohort uniquely explored the intergenerational transmission of income disparity in school readiness by delineating the role of maternal mental health symptoms in the foetal and postnatal milieu. Consistent with prior literature, lower household income predicted higher antenatal mental health problems and poorer readiness for school in the offspring (Petterson and Albers, 2001; Kiernan and Huerta, 2008; Allen *et al*., 2014; Meaney, 2018). While the effect of household income on a diverse variety of child outcomes could not be disputed, this study supported accumulating evidence that subclinical maternal depressive and anxiety symptoms, not only clinical disorders, adversely influenced children's capabilities in a graded fashion. Our two critical findings were that almost 40% of women reported high antenatal mental health symptoms and these symptoms independently explained 13–44% of the income-school readiness gap.

### Strengths of the study

LGCMs are considered a powerful approach to test for intra-individual changes over time and inter-individual differences in trajectories across time (Hertzog and Nesselroade, 1987; Curran and Hussong, 2003). In this study, while the mean mental health trajectory showed slight improvements over time, trajectories differed based on each mother's mental health in pregnancy; mothers with worse symptoms during pregnancy improved less in mood over time, when compared to those with better symptoms during pregnancy. Recent data demonstrated that earlier onset of mental health symptoms during pregnancy was a characteristic of pregnant women with moderate and severe anxiety, depression, whereas later onset (i.e. third trimester) tended to be milder (Putnam *et al*., 2017). Furthermore, predictors of severe pregnancy depression and anxiety, such as low self-esteem, poor perceived support, adverse life experiences and a history of child abuse, could potentially predispose women to parental styles characterised by low care and high control, impaired mother–infant bonding, as well as other psychiatric conditions (Leigh and Milgrom, 2008; Grant *et al*., 2012; Huizink *et al*., 2017). Thus, high severity of mental health symptoms during pregnancy likely warrants effective treatment before a cascade of downstream complications incur.

The use of symptom scale measures of depression and anxiety allowed us to compare mothers with subthreshold and clinical levels of symptoms of mothers. The association between high, subsyndromal levels of symptoms and child outcomes implied that the risk for worse child outcomes was not limited to women with clinical levels of symptoms. Rather, maternal mental health variations have negative effects on the outcomes of the next generation in a dose–response fashion. This study adds to the literature by suggesting that there are other considerable consequences to the offspring of mothers with varying levels of mental health symptoms, not only in behavioural and socio-emotional functions, but also multiple components of school readiness. This finding is of considerable public health significance as a large percentage of women face subclinical levels of affective symptoms during pregnancy.

### Limitations of the data

Although the LGCMM allowed us to examine in-depth maternal mental health trajectories from the antenatal to the postnatal period, the sample size was not large enough to test more than one mediating pathway between household income and child outcomes, as well as potential effects of other confounders. The pertinent confounders in our multi-ethnic population included parenting styles and quality, roles of father, social and family values, such as cultural expectations and incremental beliefs. To address this, we conducted sensitivity analyses to account for unmeasured confounder bias. Another limitation was that we were unable to explore the bidirectional effects of the child on maternal symptoms; however, the mother's mental health trajectory over time in the growth curve analyses likely captured the complex interplay between the child and the mother. Finally, the child behavioural and social–emotional rating scales were completed by mothers and there were previous studies showing a small association between higher maternal depression and parental tendency to over-report child behavioural problems while others did not find a ‘depressed mother reporter bias’ (Fergusson *et al*., 1993; Boyle and Pickles, 1997). Therefore, when the mother was the only source of information on the child, the association between maternal mental health and child outcomes could be stronger than in reality. In this cohort, we collected information on the child's behaviours from the fathers and our ongoing study will explore the differences between maternal and paternal reports.

### Interpretation

Similar to other studies, we confirmed stronger effects of antenatal maternal mental health on the child compared to perinatal mental health variations across time (Evans *et al*., 2001, 2012; Matthey *et al*., 2006; Monk *et al*., 2013; Qiu *et al*., 2013; Glover, 2014; O'Donnell *et al*., 2014; Rifkin-Graboi *et al*., 2015; Sandman *et al*., 2015; Lebel *et al*., 2016; Meaney, 2018). The apparent influence of antenatal maternal mental health on child outcomes suggests that maternal mental health alters the in utero environment with an impact on the foetus' brain development. This pathway may thus serve as a mechanism underlying socio-economic disparities in cognition and achievement. Our group previously used neonatal neuroimaging to demonstrate a link between antenatal maternal affective symptoms and the development of infant brain structures (Qiu *et al*., 2013; Rifkin-Graboi *et al*., 2015; Wen *et al*., 2017). In these studies, antenatal maternal symptoms of depression and anxiety were associated with the hippocampal growth and connectivity of the amygdala with the insula (Qiu *et al*., 2013; Wen *et al*., 2017).

## Conclusions

This study aims to elucidate the contribution of maternal mental health to income disparities of school readiness outcomes. While this study suggests the antenatal period as a potential time for maternal intervention, there is important evidence for the reversibility of the effects of poorer maternal mental health through postnatal intervention (Weissman *et al*., 2015; Handley *et al*., 2017; Meaney, 2018). The brain plasticity of children also allows for sensitivity to interventions in early childhood (Cicchetti *et al*., 2000; Shaw *et al*., 2009; Shonkoff *et al*., 2012; Noble *et al*., 2015). Hence, maternal mental health, whether antenatal or postnatal, may be a salient prevention focus moving forward. This study provides justifications for the need to embed universal mental health screens at the first obstetric appointment, offer low-resource therapeutic approaches for women with subthreshold depression and anxiety symptoms (e.g. web-based programs), and consider population-level prevention through improving the mental health literacy of women during early childbearing ages (Ashford *et al*., 2016; Khanlari *et al*., 2019). A novel, pre-emptive strategies to improve mother's mental health through a life-course development approach may be indispensable for optimising the achievement, cognition and behaviours of the next generation.

## Data Availability

The cohort investigators are in the process of moving towards an open cohort. In the interim, data can be shared upon request. Requests can be emailed to the corresponding author, Dr Evelyn Law at, evelyn_law@nuhs.edu.sg

## Supplementary material

For supplementary material accompanying this paper visit https://doi.org/10.1017/S204579602000102X. click here to view supplementary material
