# Supplementary material for: Income disparity in school readiness and the mediating role of perinatal maternal mental health: a longitudinal birth cohort study
Source: Epidemiol Psychiatr Sci. 2021 Jan 8;30:e6. doi: 10.1017/S204579602000102X (PMC8057379; doi:10.1017/S204579602000102X)
Supplement: Supplementary file 1 [file S204579602000102Xsup001.docx]

**Supplementary materials**

**Repeated-measures analyses**

There are different ways to conduct repeated-measures analyses. While manifest (non-latent) autoregressive models over three time points is one way to describe changes in depression and anxiety, the measurement errors in the State-Trait Anxiety Inventory (STAI) and the Edinburgh Postnatal Depression Scale (EPDS) cannot be explicitly accounted for in these models, therefore biasing the path coefficients and their standard errors. Other conventional repeated-measures analyses, such as multivariate analysis of covariance (MANCOVA), can describe an individual’s mental health trajectory; however, they fail to capture inter-individual differences in these trajectories over time. In this study, STAI and EPDS were repeated three times and there were multiple school readiness outcomes; hence, latent growth curve analysis was warranted.

**Maternal mental mealth construct**

We first tested 1) whether anxiety and depression loaded equally to one construct, 2) whether the maternal mental health construct held over time, and 3) whether mental health trajectories were different between mothers. Since mental health was measured by two tools on three occasions: first at 26 to 28 weeks of pregnancy, then 6 months later at postnatal 3-months (Time 1), and then 21 months later at postnatal 24-months (Time 2), we adjusted for spacing between time points, which was at a ratio of 6-to-21, or 1-to-3.5. To ease the interpretation, we used the “INTERCEPT” to represent individual differences in antenatal mood and the “SLOPE” as the change of mood over time.

In the Latent State (LS) models, we tested whether the two measurement tools loaded highly to the mental health constructs (Fig. S1). We found that EPDS loaded slightly better (0.84–0.93) than STAI (0.80–0.87) onto the constructs, although both instruments were highly representative of maternal mental health (*p* < 0.001). The unrestricted LS model showed a mediocre fit with χ^2^ = 28.79, *df* = 6, *p* = <0.001; RMSEA = 0.06, CFI = 0.99, and SRMR = 0.01 (Table S1).

We then tested for indicator specificity of EPDS and STAI to see whether residuals of a specific tool (i.e. EPDS) correlated more with themselves over time and whether there was specific variance not shared with another tool (i.e. STAI). An autocorrelated LS model was used (Fig. S2) and we found correlations between the residuals of the same tools over time (i.e. measurement errors of EPDS became more correlated with itself and less with STAI over time). This model’s fit statistics (χ^2^ = 6.46, *df* = 2, *p* = 0.040; RMSEA = 0.04, CFI = 1.00, SRMR = 0.01) were better compared to the unrestricted LS model. The LS model with indicator-specific (IS) factor (Fig. S3) was more superior because the IS factor represented the part of the reliable variance of anxiety that was not shared with depression and we did this by explicitly setting the correlation of the IS factor and the three Maternal Mental Health constructs to zero. This model had the best fit (χ^2^ = 2.88, *df* = 5, *p*=0.718; RMSEA <0.001, CFI = 1.00, SRMR = 0.01) compared to the unrestricted and the autocorrelated model.

**Initial Latent Growth Curve Models (LGCM)**

We then performed LGCM to model the mental health trajectory over time (Fig. S4). We again saw that both depression and anxiety loaded highly (EPDS 0.83-0.90 and STAI 0.71-0.83) to the maternal mental health construct at each time point. Antenatal Maternal Mental Health, Time 1, and Time 2 showed moderately high correlations across time (0.79 ≤ *r* ≤ 0.94, 16.68 ≤ *z* ≤ 2.53), indicating stability of intra-individual differences in maternal mood over time. The R-squared, which estimated the reliabilities of the reported symptoms on the two different questionnaires, ranged from 0.594 and 0.814 and were reasonably high, reflecting that the repeated measures allowed for precise measurements.

**Table S1**. The model fit of Latent State and Latent Growth Curve models suggesting the superiority of a solution with indicator-specific (IS) factor over competing models

| **Model** | **LL** | **χ^2^** | **AIC** | **No. of Parameter** | | **RMSEA** | **CFI** | **SRMR** |
| --- | --- | --- | --- | --- | --- | --- | --- | --- |
| **Latent State Model (LS)** | | | | | | | | |
| Unrestricted LS model | -15301.22 | 28.79  df = 6 | 30673.23 | | 21 | 0.057 | 0.990 | 0.014 |
| Autocorrelated LS model | -15301.22 | 6.46  df = 2 | 30658.90 | | 25 | 0.044 | 0.998 | 0.007 |
| LS model IS Factor | -14306.93 | 2.88  df = 5 | 28661.13 | | 22 | <0.001 | 1.000 | 0.011 |
| **Latent Growth Curve Model (LGCM)** | | | | | | |  |  |
| LGCM with IS Factor | -14306.93 | 31.27  df = 10 | 28682.56 | | 17 | 0.043 | 0.986 | 0.029 |
| LGCM with IS, predictors and outcomes | -19792.72 | 126.50  df = 63 | 39819.45 | | 54 | 0.031 | 0.980 | 0.034 |
| Footnote: AIC: Akaike Information Criterion; RMSEA: Root mean square error of approximation; CFI: Comparative fit index; SRMR: Standardised root mean square residual | | | | | | | | |

**Fig. S1**. Latent State model without measurement invariance restriction

0.64^**^

0.74^**^

0.72^**^

D_P_

A_P_

Ɛ = 0.30^**^

D_1_

A_1_

D_2_

A_2_

0.14^**^

0.87^**^

0.93^**^

0.91^**^

0.86^**^

0.80^**^

0.84^**^

0.25^**^

0.27^**^

0.17^**^

0.36^**^

Footnote: ^**^*p* < 0.01; D: Depressive symptoms from EPDS, A: Anxiety symptoms from STAI, P: Pregnancy, Time 1: 3-month postnatal, Time 2: 24-months postnatal; Ɛ: measurement error. A double-headed curved arrow shows covariance between two latent variables, as denoted by ovals. Rectangular boxes represent observed variables.

**Fig. S2.** Autocorrelated Latent State model

0.63^**^

0.71^**^

0.71^**^

D_P_

A_P_

Ɛ = 0.24^**^

D_1_

A_1_

D_2_

A_2_

0.94^**^

0.82^**^

0.95^**^

0.85^**^

0.77^**^

0.87^**^

-0.17

-0.13

0.14

0.25**

0.28^**^

0.33^**^

0.09

0.41^**^

0.11^*^

Footnote: ^*^*p* < 0.05; ^**^*p* < 0.01; D: Depressive symptoms, A: Anxiety symptoms, P: Pregnancy, Time 1: 3-month postnatal, Time 2: 24-months postnatal; Ɛ: measurement error. A solid double-headed curved arrow shows the covariance between latent variables. A dashed double-headed curved arrow shows the correlation of the residuals between observed variables.

**Fig. S3.** Latent State model with indicator-specific (IS) factor

0.61^**^

0.71^**^

0.69^**^

D_P_

A_P_

Ɛ = 0.41^**^

D_1_

A_1_

D_2_

A_2_

0.89^**^

0.83^**^

0.84^**^

0.80^**^

0.90^**^

0.72^**^

0.22^**^

0.25^**^

0.31^**^

0.19^**^

0.21^**^

0.32^**^

0.27^**^

0.28^**^

Footnote: ^**^*p* < 0.01; D: Depressive symptoms, A: Anxiety symptoms, P: Pregnancy, Time 1: 3-month postnatal, Time 2: 24-months postnatal; Ɛ: measurement error. A solid double-headed curved arrow shows the covariance between latent variables. A dashed double-headed curved arrow shows the correlation of the residuals between observed variables.

**Fig. S4.** Latent Growth Curve Model with Indicator-specific (IS) factor

-0.17*

0.79**

0.94**

0

0.84**

0.24*

0.80**

D_P_

A_P_

Ɛ = 0.31**

D_1_

A_1_

0.19*

D_2_

A_2_

0.71**

0.83**

0.89**

0.80**

0.90**

0.83**

0.20**

0.22**

0.27**

0.41**

0.29**

0.29**

0.30**

Footnote: ^*^*p* < 0.05; ^**^*p* < 0.01; D: Depressive symptoms, A: Anxiety symptoms, P: Pregnancy, Time 1: 3-month postnatal, Time 2: 24-months postnatal; Ɛ: measurement error. A solid double-headed curved arrow shows the covariance between latent variables. A dashed double-headed curved arrow shows the correlation of the residuals between observed variables.

**Figure S5.** Sensitivity plots to test the strength of any unmeasured mediator-to-outcome confounding (x = rho) that obliterates the indirect effects via Mental Health Intercept (y = 0) in the LGCMM; A) Behavioral and social-emotional problem Z-score; B) Pre-academic skills; c) IQ Z-score; d) Working memory total errors


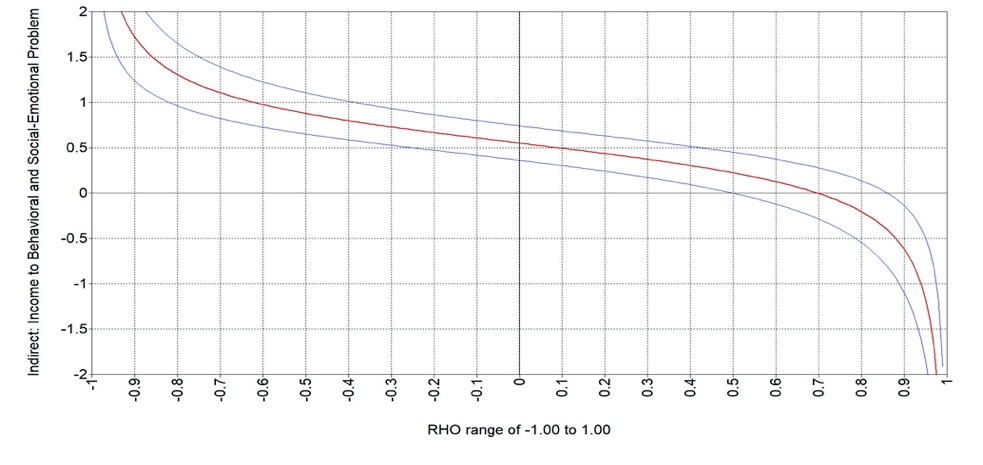


A

Indirect: Income to Behavioral and Social-Emotional Problem


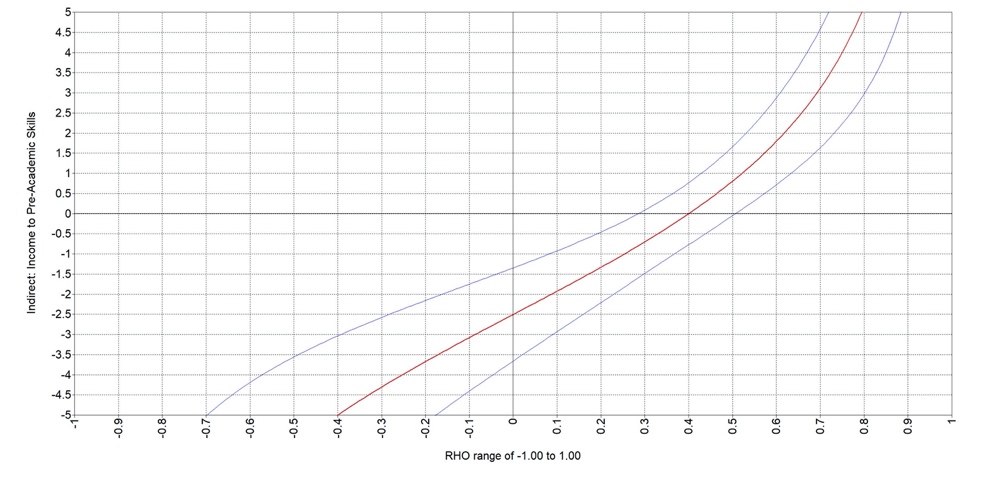


B
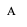


Indirect: Income to Pre-Academic Skills


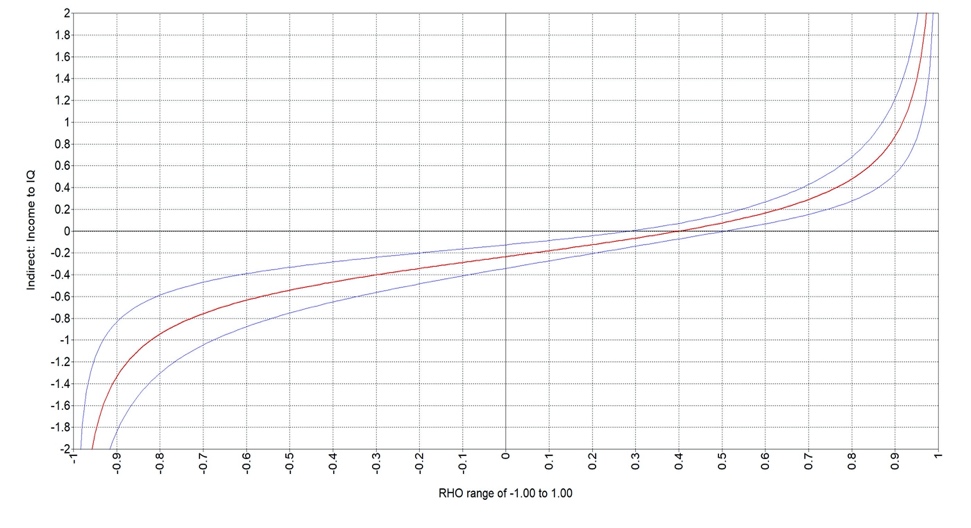


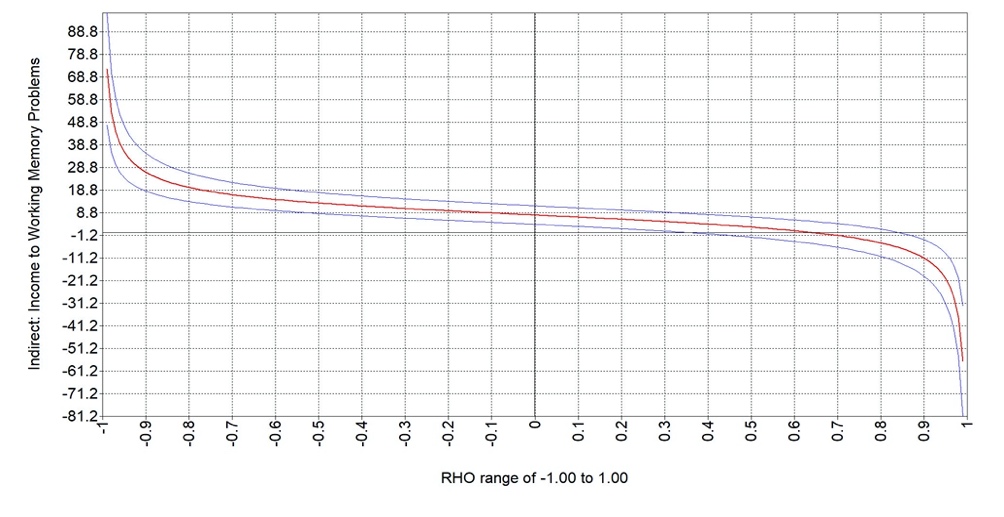


D

Indirect: Income to Working Memory Total Errors

C

Indirect: Income to IQ
